# Targeted *RP9* ablation and mutagenesis in mouse photoreceptor cells by CRISPR-Cas9

**DOI:** 10.1038/srep43062

**Published:** 2017-02-20

**Authors:** Ji-Neng Lv, Gao-Hui Zhou, Xuejiao Chen, Hui Chen, Kun-Chao Wu, Lue Xiang, Xin-Lan Lei, Xiao Zhang, Rong-Han Wu, Zi-Bing Jin

**Affiliations:** 1 Lab for Stem Cell & Retinal Regeneration, Institute of Stem Cell Research, The Eye Hospital of Wenzhou Medical University, The State Key Laboratory Cultivation Base and Key Laboratory of Vision Science, Ministry of Health Wenzhou 325027, China; 2 Division of Ophthalmic Genetics, The Eye Hospital of Wenzhou Medical University, Wenzhou 325027, China

## Abstract

Precursor messenger RNA (Pre-mRNA) splicing is an essential biological process in eukaryotic cells. Genetic mutations in many spliceosome genes confer human eye diseases. Mutations in the pre-mRNA splicing factor, *RP9* (also known as PAP1), predispose autosomal dominant retinitis pigmentosa (adRP) with an early onset and severe vision loss. However, underlying molecular mechanisms of the *RP9* mutation causing photoreceptor degeneration remains fully unknown. Here, we utilize the CRISPR/Cas9 system to generate both the *Rp9* gene knockout (KO) and point mutation knock in (KI) (*Rp9*, c.A386T, P.H129L) which is analogous to the reported one in the retinitis pigmentosa patients (*RP9*, c.A410T, P.H137L) in 661 W retinal photoreceptor cells *in vitro*. We found that proliferation and migration were significantly decreased in the mutated cells. Gene expression profiling by RNA-Seq demonstrated that RP associated genes, *Fscn2* and *Bbs2*, were down-regulated in the mutated cells. Furthermore, pre-mRNA splicing of the *Fscn2* gene was markedly affected. Our findings reveal a functional relationship between the ubiquitously expressing *RP9* and the disease-specific gene, thereafter provide a new insight of disease mechanism in *RP9*-related retinitis pigmentosa.

Retinitis pigmentosa (RP [MIM 268000]) is a group of retinal degenerative disorders with high heritability and heterogeneity, affecting approximately 1 in 4,000 in dividuals^1^,^2^ and it is becoming the leading cause of irreversible midway blindness worldwide. In RP, progressive decline of retinal function leads to night blindness, peripheral vision loss, eventually completely loss of vision in advanced stage^3^. To date, at least 27 genes have been identified to associate with the adRP^4^,^5^. Among them, many genes are preferentially or exclusively expressed in the neuro-retina and/or retinal pigment epithelium. Notably, a group of spliceosome genes, including *PRPF3*^6^, *PRPF8*^7^, *PRPF31*^8^, *RP9*^9^, *SNRNP200*^10^, *PRPF6*^11^, and *PRPF4*^12^, are ubiquitously expressed and involved in the pre-mRNA splicing process.

Pre-mRNA is crucial for the posttranscriptional regulation in mammalian gene expression. Pre-mRNA splicing reaction occurs in spliceosome which is composed of 5 small nuclear ribonucleoproteins (snRNPs), U1, U2, U4/U6, U5, and a large number of accessory protein factors^13^. The U4/U6-U5 tri-snRNP is a dynamic complex, of which structural rearrangements are critical for assembly as well as catalytic activation in the spliceosome. *RP9* has been proven to affect the assembly and/or disassembly of tri-snRNP^11^. Graziotto *et al*. and Farkas *et al*. demonstrated that *PRPF3, PRPF8* and *PRPF31* mutations could cause dysfunction of RPE^14^,^15^. Interestingly, the *RP9* caused adRP is classified as the type 2^16^, which refers to parallel deterioration of both rod and cone function in the early stage of the disease^9^. However, underlying molecular mechanisms of the *RP9* mutation causing photoreceptor degeneration remains fully unknown due to lack of appropriate disease model *in vitro* or *in vivo*.

Gene editing technology has been dramatically developed to target any genes. Clustered regularly interspaced short palindromic repeats (CRISPR) and CRISPR-associated nuclease 9 (Cas9)^17^,^18^, are the ideal way to generate *in vitro* mutational model. In CRISPR/Cas9 system, a short guide RNA (gRNA) contains about a 20 nt sequence and is capable of recognizing the targeted site followed by a protospacer adjacent motif (PAM) which recruits Cas9 to the targeted genome and induces the formation of site-specific double-stranded breaks (DSBs). The DSBs can be repaired by non-homologous end joining (NHEJ), leaving random insertions and deletions (indels), or by homology-directed repair (HDR), resulting in precise genome editing. Previous studies have shown that this system could modify genome editing in eukaryotic cells with high efficiency^19^,^20^,^21^,^22^,^23^,^24^.

Herein, we successfully generated *Rp9* gene knockout (*Rp9*-KO) and point mutation knock in (*Rp9*-KI) (*Rp9*, c.A386T, P.H129L) which was analogous to the reported one in the retinitis pigmentosa patients (*Rp9*, c.A410T, P.H137L) in 661 W retinal photoreceptor cells by using CRISPR/Cas9-mediated approach. Based on this cell model, we observed significant change in cell property and down regulation of RP associated genes, *Fscn2* and *Bbs2*. Furthermore, we elucidated that pre-mRNA splicing of the *Fscn2* gene was remarkably reduced in the mutated cells. Our study for the first time revealed a functional relationship between *Rp9*, the general splicing factor, and *FSCN2*, the photoreceptor-specific gene, and provided a new insight of disease mechanism in *Rp9*-causing retinitis pigmentosa.

## Results

### Validation of cone photoreceptor-specific markers in 661 W Cells

**Figure 1:**
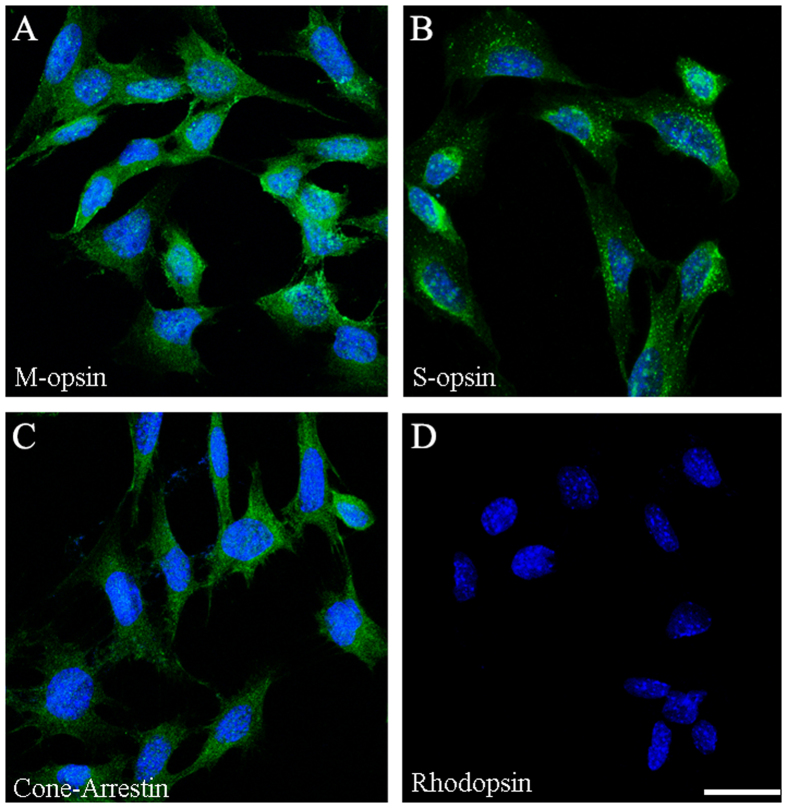
Immunocytochemical staining of 661 W cells. Cells grew on glass cover slips and were fixed with cold 4% PFA, immunolabeled with primary antibodies against red/green opsin (**A**), blue opsin (**B**), cone arrestin (**C**) and rhodopsin (**D**). DAPI (*blue*) was used to detect the nuclei. Scale bar: 25 μm.

To test 661 W cell line whether is rod photoreceptor or cone photoreceptor, cells were immunostaining for rhodopsin, blue opsin, red or green opsin and cone arrestin. The results showed that 661 W cells were positive with the cone photoreceptor-specific markers, red or green opsin (Fig. 1A), blue opsin (Fig. 1B) and cone arrestin (Fig. 1C), but negative with rod photoreceptor-specific marker, rhodopsin (Fig. 1D). These results verified that 661 W cells were cone photoreceptor originated.

### Site-specific DNA cleavage executed by Cas9/sgRNA in 661 W cells

**Figure 2:**
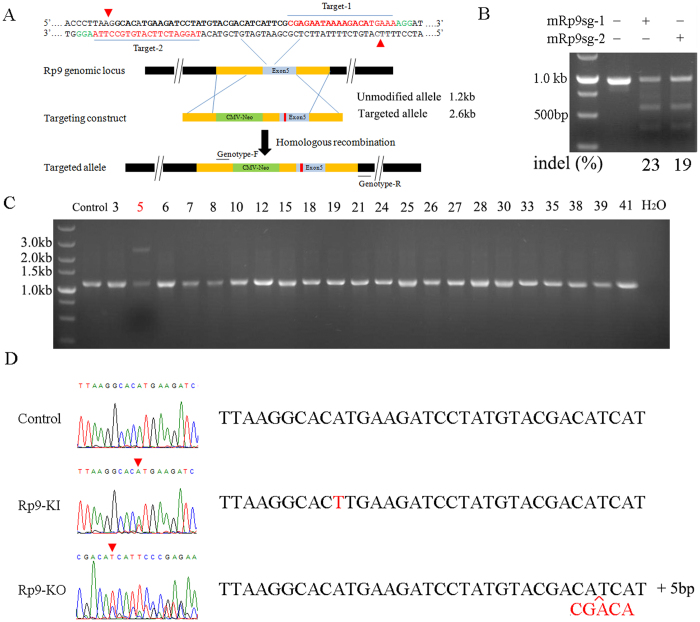
Genome editing via the type II CRISPR system in 661 W cells. (**A**) Schematic illustrating Cas9n double nicking the mouse *Rp9* locus and strategy of Cas9n pairs- mediated HDR. m*Rp9*sg-1 and m*Rp9*sg-2 sequences are shown in red. Representative cleavage sites are shown by a red triangle for 661 W cells transfected with Cas9n pairs matching target-1 and 2. The targeting vector includes homology arms (HA) flanking a CMV-Neomycin element and *Rp9* point mutation. (**B**) SURVEYOR assay for Cas9 and sgRNA-mediated indels. 661 W cells were transfected by empty vectors or vectors expressing m*Rp9*sg-1 or m*Rp9*sg-2 to test cutting efficiency in the endogenous *Rp9* locus. The DNA in the specific cells mentioned above were extracted and performed by PCR and T7EI assays. The percentage of indels was quantified by the ImageJ software. (**C**) PCR genotyping. As shown, 1 clone (marked with red number) out of 22 randomly selected clones had the expected *Rp9* mutation insertion. (**D**) Representative Sanger sequencing results of the PCR amplicons from 22 clones showing point mutation and insertion (red).

To test whether Cas9/sgRNA could cleave targeted regions of *Rp9*, we designed two sgRNAs targeting different regions of *Rp9* gene (Fig. 2A). Each Cas9/sgRNA was transfected into 661 W cells and a T7EI assay was performed to determine the cutting efficiency of each sgRNA. The results indicated that sgRNA-1 and sgRNA-2 designed to target the *Rp9* gene were highly active, inducing mutations at frequencies of 23% for sgRNA-1 and 19% for sgRNA-2 (Fig. 2B). Furthermore, no mutation was detected using the T7EI assay when cells were transfected with Cas9 plasmid alone. Taken together, these results suggested that sgRNA-1 and sgRNA-2 efficiently targeted the *Rp9* and led to NHEJ-mediated indels at target sites.

### Generation of *Rp9*-KI and *Rp9*-KO in 661 W cells

Encouraged by the high efficiency of Cas9/sgRNAs, we explored whether these Cas9n pairs could catalyze site-specific DNA cleavage and HDR in 661 W cells. 661 W cells were transfected with donor vector and Cas9n pairs targeting the exon 5 of *Rp9* gene. 3days later, transfected cells were selected for further 7 days with 1.0 mg/ml G418. After selection, cells were digested and 500 cells were transferred to a 10-cm dish. 2 weeks later, clones were picked out and propagated for another week. After propagation, 22 clones were randomly selected for genotyping. The genomic DNA of 22 clones was analyzed by PCR amplification with specific primers showed as Supplemental Table 1. Figure 2C illustrated that clone 5 contained two different amplification products whereas other clones contained only one amplification product. Sequencing result of the upper bands of clone 5 revealed the point mutation, c.A386T (Fig. 2D). The result demonstrated that the clone 5 was a heterozygous *Rp9*-KI cell. Also, we sequenced the other 21 clones, the results showed that five clones were heterozygous *Rp9*-KO cells which only one allele was mutated with nucleotide indels thus causing a frameshift mutation. Thus Clone 12 would represent for *Rp9*-KO (Fig. 2D).

### Decreased proliferation and migration by *Rp9*-KI and *Rp9*-KO

**Figure 3:**
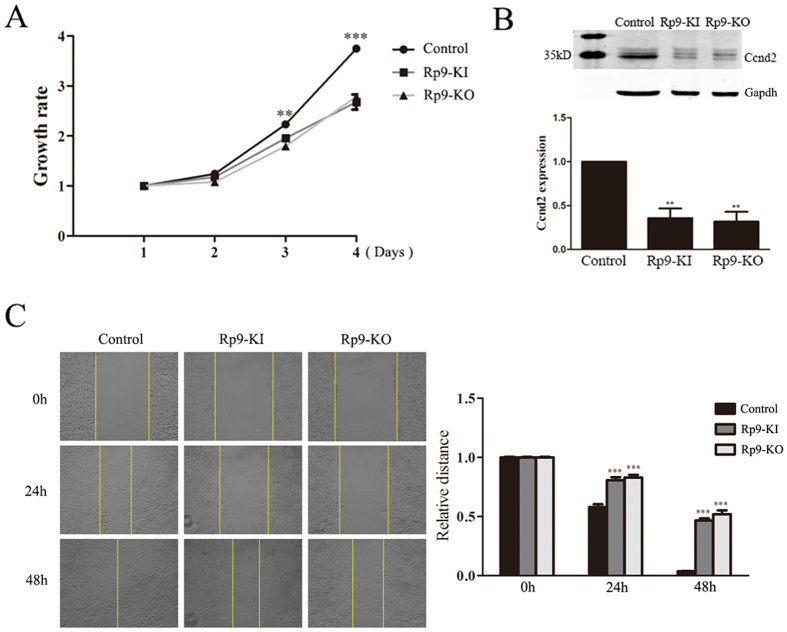
*Rp9* gene mutation inhibited the proliferation and migration of 661 W cells. (**A**) MTT cell proliferation assay was performed at indicated days. Data at each time point were expressed as mean ± SEM based on results obtained from triplicates. **P < 0.01, ***P < 0.001. (**B**) *Rp9* gene mutation down-regulated Ccnd2 expression. 661 W, *Rp9*-KI and *Rp9*-KO cell lysate were prepared and used for Western blot analysis. Gapdh was used as an internal loading control. The band intensity was measured with ImageJ software, the fold change was normalized to the level of 661 W group. Data are representative of three independent experiments. **P < 0.01. (**C**) *In vitro* scratch assays were performed to evaluate the migration potential of 661 W, *Rp9*-KI and *Rp9*-KO cells.

We sought to detect whether these *Rp9* mutations had any biological effects on 661 W cells. *Rp9*-KI and *Rp9*-KO showed inhibition of cell growth as compared with control based on the MTT assay (Fig. 3A). A reduction in growth rate was detected at day 3. At day 4, the growth rate decreased 27.6% in *Rp9*-KI and 25.6% in *Rp9*-KO cells compared with control group (n = 3, P < 0.001, Fig. 3A). Western blot analysis confirmed that proliferation-related gene Ccnd2 expression was remarkably reduced in *Rp9*-KI and *Rp9*-KO cells (Fig. 3B). We then performed *in vitro* scratch assay to examine whether *Rp9* mutations were involved in the regulation migration of 661 W cells. A dramatic migration reduction was observed in *Rp9*-KI and *Rp9*-KO cells (Fig. 3C).

### RNA-seq and differential expression analysis of *Rp9*-KI and *Rp9*-KO cells

Three libraries were generated from 661 W, *Rp9*-KI and *Rp9*-KO groups and summaries of RNA-Seq analyses are shown in Supplement Table 3. About 42.66 (661 W), 38.56 (*Rp9*-KI), and 39.83 (*Rp9*-KO) million clean reads were obtained for each transcriptome. The Q30 scores (the average quality value) were above 93%. The RNA-Seq raw reads have been submitted to the NCBI SRA database (accession number: SRR5131155, SRR5131263, and SRR5131264).

**Figure 4:**
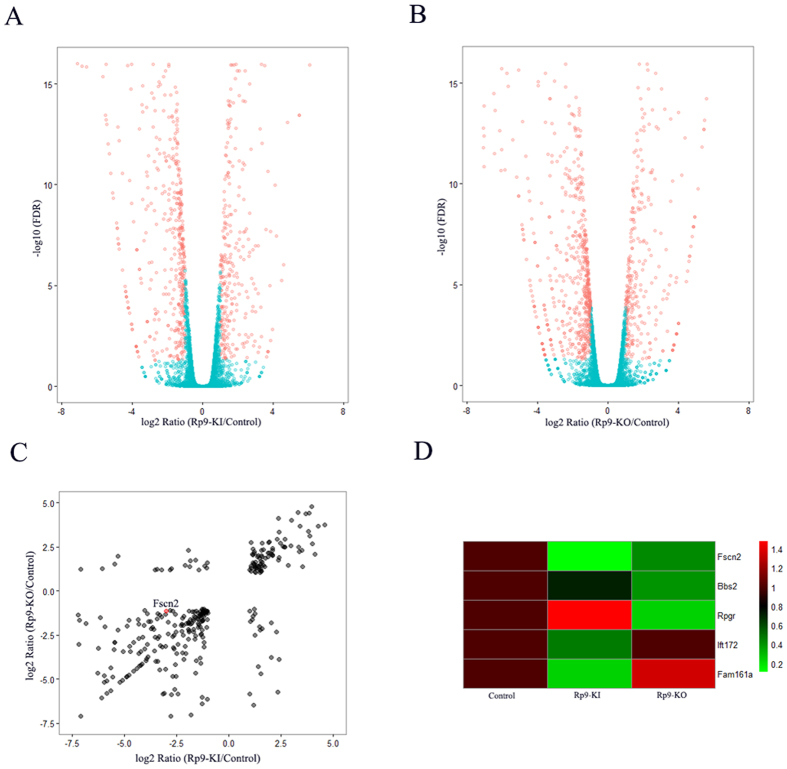
RNA-seq analyses detected gene expression changes in *Rp9* mutant cells. (**A**) Volcano plot showing genes differentially expressed in *Rp9*-KI compared with control group. Red dots show the 784 genes with a |log2FC| > 1 and FDR* < *0.05. (**B**) Volcano plot showing genes differentially expressed in *Rp9*-KO compared with control group. Red dots show the 934 genes with a |log2FC| > 1 and FDR* < *0.05. (**C**) Overlap of differential gene expression between*Rp9*-KI and *Rp9*-KO group. Red dots show the *Fscn2* gene down-regulated in both groups. (**D**) Heatmaps present RP associated gene expression in *Rp9*-KI and *Rp9*-KO group.

**Figure 5:**
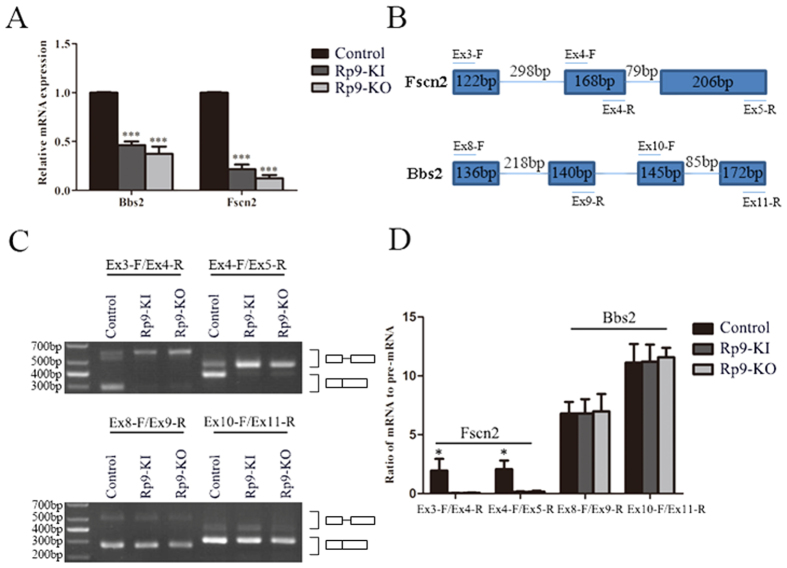
*Rp9* gene mutation significantly affects *Fscn2* pre-mRNA splicing. (**A**) qRT- PCR analysis of *Fscn2* and *Bbs2* transcripts in 661 W, *Rp9*-KI and *Rp9*-KO cells. Relative expression levels of mRNA were normalized against *Gapdh*. ***P < 0.001. (**B**) Genomic structures of *Fscn2* and *Bbs2* genes and primers used for RT-PCR. (**C**) Effects of *Rp9* gene mutation on the pre-mRNA splicing of *Fscn2* intron 3, intron 4 and *Bbs2* intron 8, intron 10. RT-PCR was used to detect the splicing products and corresponding pre-mRNA using specific primers as depicted in panel B. (**D**) Quantification of *Fscn2* and *Bbs2* splicing efficiency by measuring the ratio of mRNA to pre-mRNA using ImageJ from three independent experiments. *P < 0.05.

Through RNA-Seq, a total of 14006, 13885 and 13226 expressed genes were detected in 661 W, *Rp9*-KI and *Rp9*-KO cells respectively. Among them, 784 and 934 genes were differentially expressed in *Rp9*-KI and *Rp9*-KO cells compared with 661 W cells (Fig. 4A,B). Overlap of differentially expressed genes containing down-regulated RP associated gene, *Fscn2*, identified by *Rp9*-KI and *Rp9*-KO cells were presented (Fig. 4C). We used qRT-PCR to confirm the expression of *Fscn2* and found it to be markedly down-regulated in *Rp9*-KI and *Rp9*-KO cells (Fig. 5A). We also used qRT-PCR to quantify other RP associated genes which were significant down-regulated in *Rp9*-KI or *Rp9*-KO cells (Fig. 4D) and found another gene, *Bbs2*, also significantly decreased in *Rp9-KI* and *Rp9-KO* cells (Fig. 5A).

### *RP9* mutagenesis affects pre-mRNA splicing of photoreceptor gene *FSCN2*

Previous report showed that *PRPF31* mutation inhibited splicing of retina-specific gene, *RHO*^25^. In order to confirm whether *RP9* mutation inhibited retina-specific splicing substrate genes expression, we selected *Fscn2* and *Bbs2* for the further experiments. *Fscn2* and *Bbs2* pre-mRNA splicing were examined using RT-PCR with specific primers (Fig. 5B, Supplemental Table 1). As shown in the Fig. 5C, *Fscn2* splicing was significantly inhibited in *Rp9*-KI and *Rp9*-KO compared with control group. Quantification of splicing efficiency obtained from three independent experiments was shown in Fig. 5D. However, *Rp9*-KI and *Rp9*-KO did not affect the *Bbs2* splicing, because there was no significant change in the ratio of pre-mRNA in splicing products (Fig. 5D).

## Discussion

We have demonstrated that *RP9* patient-specific rod photoreceptors conferred degeneration *in vitro* by using patient-specific induced pluripotent stem cells^26^. However, underlying molecular mechanisms of the *RP9* mutation causing photoreceptor degeneration remains unknown. Pre-mRNA splicing is crucial for the posttranscriptional regulation of gene expression, providing significant expansion of the functional proteome of eukaryotic organisms with limited genes^27^. Mutations that interfere with splicing play an important role in human eye disease^28^,^29^,^30^. Here we have shown that proliferation and migration significantly decrease in the *Rp9* mutant 661 W photoreceptor cells. RP associated genes, *Fscn2* and *Bbs2*, both are markedly down-regulated in the mutated cells. Further investigation indicated that pre-mRNA splicing of the *Fscn2* gene was markedly reduced.

In this study, we successfully generated *Rp9*-KI (HDR) with an efficiency of 4.5% and *Rp9*-KO (Indels) with an efficiency of 22.7%, which was consistent with reports from other groups. For example, Mali *et al*. reported 10–25% indel rates in 293 T cells in 2013^20^. Likewise, in mouse embryonic stem cells study, the efficiency of NHEJ-mediated knock-out was 28–50%, whereas the efficiency of HDR-mediated knock-in was below 10%^23^. Importantly, *Rp9*-KI and *RP9*-KO resulted in inhibition of cell proliferation and migration.

Previous work by Yuan *et al*. demonstrated that *PRPF31* mutation could inhibit *RHO* splicing^25^. Through RNA sequencing, we confirmed the expression of *Fscn2* markedly down-regulated in *Rp9*-KI and *Rp9*-KO cells. Fscn2, actin-bundling proteins, is a photoreceptor-specific protein of fascin family, which plays an important role in photoreceptor disk morphogenesis. A frame-shift mutation in *FSCN2*, 208delG, was reported in Japanese adRP patients^31^. A mouse model carrying a targeted disruption of Fscn2 showed progressive photoreceptor degeneration^32^.

RT-PCR results showed that the 661 W cell had normal pre-mRNA splicing of *Fscn2*, whereas *Rp9*-KI and *Rp9*-KO cells inhibited this splicing progress. Our results were consistent with those recently reported by Mordes *et al*., who found dominant-negative effect of *PRPF31* mutation on *FSCN2* pre-mRNA splicing^33^. However, Maita *et al*. reported that *RP9* p.H137L mutation had no effect on E1A splicing^34^. This might suggest that *Rp9* only inhibits a fraction of gene splicing. Our experiments revealed a functional link between ubiquitously expressed *Rp9* gene and the expression of the retina-specific gene, *Fscn2*. Our results that *Rp9*-KI and *Rp9*-KO blocking pre-mRNA splicing of *Fscn2* provided a new insight for the photoreceptor-specific phenotype of *RP9* mutations. Defected *Fscn2* gene products may contribute to photoreceptor cell death.

We also detected the RP associated gene, *Bbs2*, markedly down-regulated in *Rp9*-KI and *Rp9*-KO cells. Our result showed that *Rp9*-KI and *Rp9*-KO did not affect the *Bbs2* splicing. It demonstrated that not all splicing events were equally sensitive to *Rp9* mutation. *Rp9* mutants may inhibit pre-mRNA splicing of a subset of photoreceptor genes, such as *Fscn2* intron 3 and intron 4, but not other splicing events such as *Bbs2* intron 8 and intron 10.

In summary, we have shown that *Rp9* mutations obviously inhibit cell proliferation and migration. Furthermore, the splicing of adRP associated gene, *Fscn2*, is also significantly inhibited in *Rp9* mutation cells. Our results on RP9 provide an explanation why the mutations in the ubiquitously expressed splicing factors can cause adRP.

## Materials and Methods

### Donor vectors and sgRNA

To construct donor templates, *Rp9* fragments and CMV-Neo were amplified with Phanta Max Super-Fidelity DNA Polymerase (Vazyme Biotech Co.Ltd, Nanjing, China) using gene-specific primers (Supplemental Table 1). *Rp9* fragments were cloned into pEASY-Blunt Simple Cloning Vector (pEASY-*Rp9*), and then CMV-Neo was cloned into SacI sites of pEASY-*Rp9* (Supplemental Table 2). sgRNAs of m*Rp9* were ordered, annealed, phosphorylated and cloned into the BbsI-digested Cas9 nickase vector (pX335, Addgene plasmid ID: 42335) and Cas9 vector (pX330, Addgene plasmid ID: 42230).

### Cell culture

Cone-derived cell line (661 W) originated from a transgenic mouse line with retinal tumor^35^ was cultured in DMEM medium (Gibco, Carlsbad, USA) supplemented with 10% heat-inactivated FBS (Gibco, Carlsbad, USA)and 100 μg/mL penicillin/streptomycin at 37 °C in a humidified atmosphere of 5% CO_2_. 661 W cells were plated into 6-well plates for transfection. After twenty four hours, cells were replaced with new complete medium and the DNA mixed with FuGENE HD Reagent (Roche, Basel, Switzerlands) in Opti-MEM (Gibco, Carlsbad, USA) according to the manufacturer’s manual. For G418 selection, 661 W cells transfected with px335-m*Rp9*sg1, px335-m*Rp9*sg2 (Cas9n pairs) and donor vector were selected with 1 mg/ml of G418.

### Genomic DNA isolation, amplication and T7EI assay

To validate the m*Rp9* sgRNAs, the genomic DNA of each Cas9/gRNA-transfected cells was extracted using the Blood/Cultured cells DNA Kit (Simgen Biotech Co.Ltd, Hangzhou, China) following the manufacturer’s instruction. The regions containing thetarget sites were amplified by PCR using Phanta Max Super-Fidelity DNA Polymerase with gene-specific primers (Supplemental Table 1) under the following conditions: 95 °C for 3 min; 30 cycles (95 °C for 15 s, 58 °C for 15 s, 72 °C for 30 s) and 72 °C for 5 min.

The T7EI assay was performed according to the manufacturer’s instructions. In brief, 20 μl of PCR product was denatured and annealed by heating at 95 °C for 5 min and ramped down to 25 °C at 6 °C/min. Then, 5 μl annealed samples, 1.1 μl NEBuffer 2 (10x), 0.5 μl T7EI and 4.4 μl ddH_2_O were added together and incubated at 37 °C for 30 min. Cleaved DNA fragments were separated on 1.5% agarose gels and the DNA concentration of each band was quantified using the ImageJ software. Percent values of indels were calculated as described^15^. For genotyping PCR, genomic DNA was amplified using 2 × Super Taq PCR MasterMix (BioTeke Corporation, Beijing, China) with primers listed (Supplemental Table 1).

### Immunocytochemistry

661 W cells were fixed in 4% paraformaldehyde (PFA) for 15 minutes. Cells were permeabilized and blocked in PBS containing 4% BSA and 0.5% Triton X-100 for 1 hour at room temperature, incubated overnight with primary antibody at 4 °C, and then subjected to immunohistochemistry as previously described^36^.

Primary antibodies were rabbit anti-opsin blue (1:500; chemicon, AB5407), rabbit anti-opsin red/green (1:500; chemicon, AB5405), rabbit anti-cone arrestin (1:500; Millipore, AB15282) and mouse anti-rhodopsin (1:10000; sigma, o4886).

### Cell proliferation assay

A total of 5 × 10^3^ 661 W cells were seeded in 96-well plates with 100 μl DMEM containing 5% FBS and 100 μg/mL penicillin/streptomycin. Cell proliferation was assessed by MTT assay. MTT assay was carried out according to the method by Mosmann^37^. Briefly, 10 μl of MTT solution was added into each well and the cells were incubated for 4 hours. Then medium was discarded and supplied with 150 μl DMSO. Finally, Cell proliferation was assessed by measuring the absorbance at 490 nm using Spectra Max M5 (Molecular Devices, Sunnyvale, CA, USA).

### *In Vitro* scratch assay

*In Vitro* Scratch Assay was performed as previously reported^38^. Simply, cells were scratched with a 200 μl pipet tip. Remove the debris by washing the cells once with 1 ml of DPBS and then replace with 2 ml DMEM supplemented with 2% FBS and 100 μg/mL penicillin/streptomycin. Photograph was taken immediately after scratching and at 24 or 48 hours after culture. The ability of migration was evaluated by comparing with the migration rate in the center of the gap.

### Western blot analysis

Total protein extracts were prepared using cell lysis buffer containing protease inhibitors (1 mM phenylmethylsulfonyl fluoride, 2 mM leupeptin, 1 mM pepstatin, 80 mM aprotinin). Protein contents were quantified by the Bradford reagent according to the manufacturer’s instructions. Equal amounts of proteins were separated by 12% SDS-PAGE and transferred to a nitrocellulose blotting membrane (PALL Corporation, Port Washington, USA). Membranes were blocked for 1 h in 1 × TBS containing 10% non-fat milk, 0.1% Tween 20 and incubated overnight with primary antibodies: rabbit anti-Ccnd2 (Santa Cruz Biotechnology, Santa Cruz, USA), mouse anti-Gapdh (KangChen Bio-tech Inc., Shanghai, China). Then membranes were incubated with IRDye^®^ 680-conjugated goat anti- rabbit, IRDye 800CW-conjugated goat anti-mouse. Fluorophore-conjugated antibodies were detected using the Odyssey^®^ Imager (LI-COR Biosciences Inc., Lincoln, USA).

### RNA library construction and sequencing

RNA was extracted from the 661 W cells, *Rp9*-KI and *Rp9*-KO mutated cells using TRIzol reagent (Invitrogen, San Diego, USA). RNA concentration and purity were measured with NanoDrop 2000 Spectrophotometer (Thermo Fisher Scientific, Wilmington, USA). RNA integrity was assessed using the RNA Nano 6000 Assay Kit of Agilent Bioanalyzer 2100 system (Agilent Technologies, Santa Clara, USA).

High-quality RNA was sent to Biomarker Technologies Corporation (Biomarker Technologies Corporation, Beijing, China) for cDNA libraries construction and sequencing. The RNA-Seq libraries were constructed according NEBNext UltraTM RNA Library Prep Kit for Illumina (New England Biolabs, Ipswich, USA) following manufacturer’s recommendations. Briefly, mRNA was purified by NEBNext Poly (A) mRNA Magnetic Isolation Module. The isolated mRNA was fragmented and used to synthesize the first cDNA. Second strand cDNA synthesis was generated using DNA Polymerase I and RNase H. The double-stranded cDNAs were purified by Agencourt AMPure XP system (Beckman Coulter, Brea, USA) and subjected to end repair and adapter ligation. The ligation products were enriched by PCR amplification and purified using Agencourt AMPure XP system. Sequencing reactions were carried out on the Illumina HiSeq 2500.

### Transcriptome analysis and identification of differential gene expression

The raw reads were firstly processed through in-house perl scripts. Clean reads were obtained by removing reads containing adapter sequences, unknown nucleotides>5%, low quality reads. The clean reads were mapped to mouse genome (mm10) with TopHat2^39^. Gene expression levels were estimated using fragments per kilobase of exon per million fragments mapped (FPKM).

Prior to differential gene expression analysis, the read counts of each sequenced library were adjusted by edgeR program package^40^. DEGseq^41^ was performed to evaluate differential expression between control, *Rp9*-KI and *Rp9*-KO groups. The false discovery rate (FDR) control method was applied to define the threshold of the P-value to compute the level of significance. Significantly differential expression was accepted as |log2FC| > 1 and FDR < 0.05.

### RT-PCR

For reverse transcription, isolated RNA was treated with DNase I (Thermo Scientific, Rockford, USA) according to the manufacturer’s manual. The DNase I-treated RNA was transcribed into cDNA using MMLV Reverse Transcriptase (Promega, Madison, USA). The cDNA was subsequently used as template to perform RT-PCR and qRT-PCR.

RT-PCR was performed according to the following procedures: 12.5 μl 2×Super Taq PCR Master Mix, 0.5 μl cDNA, 11 μl ddH_2_O, 0.5 μl forward primer (10 μM), 0.5 μl reverse primer (10 μM) (Supplemental Table 1), reaction conditions including 95°C for 3 min; 40 cycles (95 °C for 15 s, 58 °C for 15 s, 72 °C for 30 s) and 72 °C for 5 min.

qRT-PCR was performed as described in the method of Fast start universal SYBR Green Master (Roche Molecular Biochemicals, Mannheim, Germany) with 7500 Real-Time PCR System. *Gapdh* expression was used for normalization. The specificity of PCR products was verified by melt curve analysis. Sequences of primers are shown (Supplemental Table 1).

### Statistical analysis

Data were expressed as means ± SEM and analyzed by two-way analysis of variance (ANOVA) with GraphPad. Differences were considered to be statistically significant at a P value of 0.05 or less.

## Additional Information

**How to cite this article**: Lv, J.-N. *et al*. Targeted *RP9* ablation and mutagenesis in mouse photoreceptor cells by CRISPR-Cas9. *Sci. Rep.* **7**, 43062; doi: 10.1038/srep43062 (2017).

**Publisher's note:** Springer Nature remains neutral with regard to jurisdictional claims in published maps and institutional affiliations.

## Supplementary Material

Supplementary Information
