# Supplementary material for: Targeted RP9 ablation and mutagenesis in mouse photoreceptor cells by CRISPR-Cas9
Source: Sci Rep. 2017 Feb 20;7:43062. doi: 10.1038/srep43062 (PMC5317003; doi:10.1038/srep43062)
Supplement: Supplementary Information [file srep43062-s1.pdf]

Targeted *RP9* ablation and mutagenesis in mouse photoreceptor cells by CRISPR-Cas9

Ji-Neng Lv[1,2,3], Gao-Hui Zhou[1,2,3], Xuejiao Chen[1,2,3], Hui Chen[1,2], Kun-Chao Wu[1,2], Lue Xiang[1,2], Xin-Lan Lei[1,2], Xiao Zhang[1,2], Rong-Han Wu[1,2], Zi-Bing Jin[1,2]*

1. Lab for Stem Cell & Retinal Regeneration, Institute of Stem Cell Research, The Eye Hospital of Wenzhou Medical University, The State Key Laboratory Cultivation Base and Key Laboratory of Vision Science, Ministry of Health Wenzhou 325027, China;

2. Division of Ophthalmic Genetics, The Eye Hospital of Wenzhou Medical University, Wenzhou 325027, China.

3. These authors contributed equally.

*Correspondence:

E-mail: Dr. Zi-Bing Jin, Lab for Stem Cell & Retinal Regeneration, Institute of Stem Cell Research, Division of Ophthalmic Genetics, The Eye Hospital, Wenzhou Medical University, Wenzhou 325027, China. E-mail: jinzb@mail.eye.ac.cn. Tel/fax: +86-577-88067926.

Table S1. Primer set for PCR

Gene	Primer sequences (5′ → 3′)
mRp9sg-1s	CACCGTAGGATCTTCATGTGCCTTA
mRp9sg-1a	AAACTAAGGCACATGAAGATCCTAC
mRp9sg-2s	CACCGCGAGAATAAAAGACATGAAA
mRp9sg-2a	AAACTTTCATGTCTTTTATTCTCGC
Genotype-R	GGAGTCCTCCAGTAACTG
Genotype-F/mRp9T7EI-F	CAAGCTACAGGAAGCTAACTG
mRp9T7EI-R	CCACTGCATATTTAGGGATCTAC
HomomRp9-F	ACCGGTGCTCTAGTCTATCCTGGTCT
HomomRp9-R	AGACAGAGCCACCACAGC
mRp9mutant-F	CTTAAGGCACTTGAAGATCCTAT
mRp9mutant-R	ATAGGATCTTCAAGTGCCTTAAG
CMV-SacI	GAGCTCAGTTATTAATAGTAATCAATTACGG
Neo-SacI	GAGCTCTTTATTTCAGAAGAACTCGTCAAGAAG
mFscn2-QF	CGACTTTGTGGGCGAGGAC
mFscn2-QR	CCTCCACGGCCTCTAATCTG
mBbs2-QF	AGGATGCTCGGCTGATGAGG
mBbs2-QR	GCTTGGTTCACCGCCTTCAG
mGapdh-QF	TGCGACTTCAACAGCAACTC
mGapdh-QR	CTTGCTCAGTGTCCTTGCTG
mFscn2-Ex3-F	CTCTGCCAACACCATGTTTG
mFscn2-Ex4-R	CTCTAATCTGATAGGCGCCATC
mFscn2-Ex4-F	GCGAGGACGAGCTATTTACC
mFscn2-Ex5-R	TCAGTATTCCCAGAGAGCTTC
mBbs2-Ex8-F	GTTGATGCTCGCAGTGAC
mBbs2-Ex9-R	CTTGGTGCTCTCCTCATAG
mBbs2-Ex10-F	GCAGAACTGAGCAGTCCC
mBbs2-Ex11-R	CTGCTTCTGTAACCCACG

Table S2 Homologous repair sequences used in this study

Gene name	Sequence (5 to 3)
The left homologous arm(Green)	ACCGGTGCTCTAGTCTATCCTGGTCTACAAGGAGAACTTCATGCCACTAGGCTACATAGTGAGACCCTGCCTCAAACAAA
	AAACAACAAAAAGATGCTCTGGAACTGGAGTTGTAGATGGTTGTGAGCTGTCATGTAGATGCTGAGAATAGAAACTGTCC
	TGGAAGAGCAGCGGTGCTCTACCCACTGACCCTCTTCCCAGCCCTCCCTATAAAATTGGAGAAGTGTTTTGTACCCTTAGT
	GTATCTACAGTAATAAAGTCTGAGTATGGTGGCACACAGCTATGATCTCAGTATTCAGAAGGCTGAAGCAGGATTGCTGTG
	ACTTAGGGGGCAGCTTGGTCTACATAGTGAGTTTGAGGGTCATCCAATAACTGTTTCAAAACATAATAAGACTTATCTCCA
	AAAGCCCTAATAATTATAGGCCTTTAAAGGGATTAGAATACATTGTAAGACAGGTGTGATGGTTTCTGTTTGAGAGCCCAG
	CACTTTGGAGACTGCAGCAGGATCAGCATAGTACAAAGCCATCCTGGACAGCATTGTGAGGCCTTGTCTCAAGATGAGCT
	GGACGCAGTGCTGACCCAGCACTGCAGGGTACAAGCAGGAGGGTCAAGACTTCAAGATCATCTGCAGCGCCTTAGCAAG
	TTCAAAGCTAGCCTGAGCTATATAGTAAGATCTTACATGGTTGGGAAAAGACATAGATGCTGATATATCTATCATCAAGCTA
CMV-Neo (Purple)	CAGGAAGCTAACTGTATTCTTGGCACTCAGGGGGCTAAGAAGGGAAGGTTAAAAACTCAAGGCCAGTCTGAGCTCAGTT
	ATTAATAGTAATCAATTACGGGGTCATTAGTTCATAGCCCATATATGGAGTTCCGCGTTACATAACTTACGGTAAATGGCCCG
	CCTGGCTGACCGCCCAACGACCCCCGCCCATTGACGTCAATAATGACGTATGTTCCCATAGTAACGCCAATAGGGACTTTC
	CATTGACGTCAATGGGTGGAGTATTTACGGTAAACTGCCCACTTGGCAGTACATCAAGTGTATCATATGCCAAGTACGCCC
	CCTATTGACGTCAATGACGGTAAATGGCCCGCCTGGCATTATGCCCAGTACATGACCTTATGGGACTTTCCTACTTGGCAGT
	ACATCTACGTATTAGTCATCGCTATTACCATGGTGATGCGGTTTTGGCAGTACATCAATGGGCGTGGATAGCGGTTTGACTC
	ACGGGGATTTCCAAGTCTCCACCCCATTGACGTCAATGGGAGTTTGTTTTGGCACCAAAATCAACGGGACTTTCCAAAAT
	GTCGTAACAACTCCGCCCCATTGACGCAAATGGGCGGTAGGCGTGTACGGTGGGAGGTCTATATAAGCAGAGCTGGTTTA
	GTGAACCGTCAGATCCGCTAGCGCTACCGGACTCAGATCTCGAGATGATTGAACAAGATGGATTGCACGCAGGTTCTCCG
	GCCGCTTGGGTGGAGAGGCTATTCGGCTATGACTGGGCACAACAGACAATCGGCTGCTCTGATGCCGCCGTGTTCCGGCT
	GTCAGCGCAGGGGCGCCCGGTTCTTTTTGTCAAGACCGACCTGTCCGGTGCCCTGAATGAACTGCAAGACGAGGCAGCG
	CGGCTATCGTGGCTGGCCACGACGGGCGTTCCTTGCGCAGCTGTGCTCGACGTTGTCACTGAAGCGGGAAGGGACTGGC
	TGCTATTGGGCGAAGTGCCGGGGCAGGATCTCCTGTCATCTCACCTTGCTCCTGCCGAGAAAGTATCCATCATGGCTGATG
	CAATGCGGCGGCTGCATACGCTTGATCCGGCTACCTGCCCATTCGACCACCAAGCGAAACATCGCATCGAGCGAGCACGT
	ACTCGGATGGAAGCCGGTCTTGTCGATCAGGATGATCTGGACGAAGAGCATCAGGGGCTCGCGCCAGCCGAACTGTTCG
	CCAGGCTCAAGGCGAGCATGCCCGACGGCGAGGATCTCGTCGTGACCCATGGCGATGCCTGCTTGCCGAATATCATGGTG
	GAAAATGGCCGCTTTTCTGGATTCATCGACTGTGGCCGGCTGGGTGTGGCGGACCGCTATCAGGACATAGCGTTGGCTAC
	CCGTGATATTGCTGAAGAGCTTGGCGGCGAATGGGCTGACCGCTTCCTCGTGCTTTACGGTATCGCCGCTCCCGATTCGCA
Point mutation (Red)	GCGCATCGCCTTCTATCGCCTTCTTGACGAGTTCTTCTGAAATAAAGAGCTCCATTGCCAGTTTGAAGCCAGTCCAGGATA
	AATAAAGACTCTCAAATTTAACAACAACAACAACAACAACAAAATCTACATCAAATATTAGTTTCTGTACATAGAGGTTTA
	ATTTTTACATGATAAGCAGTACATTGTAACTAACCAAACTGCAACCCTCTACAACCCTTAAGGCAC**T**TGAAGATCCTATGTA
	CGACATCATTCGCGAGAATAAAAGACATGAAAAGGATGTAAGGTAAGGCCGCTCGCTCATCGATACCAATGTCCTCTCTCT
	TGTTGTTGGGTTTTTTGTTTGTTTTTCTTTTTTTTTTTTCGAGACAGGGTTTCTTTGTGTAGCCCTAGCTGTCCTGGAACTCA
	CTCTGTAGACTAGGCTGGCCTCAAACTCAAAAATCTGCCTGCCTCTGCCTGAGTGCTGGGATTAAAGGCGTGCGCCACCA
	CGCCCGGCTCCAATGTCCTCTTGAAGTCTGAAATCATGCAATGTTATAAACAACACTGGGAGAGCAAGATTAATCACTGAA
	CAGAGATGTCAGGTAACAAGCAGGTCAGCTTCTACTGTAGATCCCTAAATATGCAGTGGGCATGCCTCGACTTGTGGACAA
	TCACATTGTGGTGTGTGTGTGTGTCTATGTGTGGTGTGTGTGGCTACAGTGTAGGCCAAGCTGGCCTCAGTCTCTGGAGTG
The right homologous arm(Black)	CTGAGATGACTCATATGCCACCACACTCTGTGTGACTGACGAAGTCCAAGTTTTTGTTATTTTCATTTTTAGACAAGATTAA
	GGAAGGGGCTGTGGTTCAGTTGGTAGCATGTCTGAAGCCCTGGGTTGTGTTGGATCCCCAGCACGGCATATACCAGCTATT
	AGGATGCACACATATAGCACCATCACTTAGGACGTGAAGGCTGGAGGATTAGAAGTCAAGGTCACGAGTGACTGGGCATG
	AGTTGAGGCTAGCCTGGAATAATGAACACCAGGAAGGTGTGTTTGCTAATTGCTGTGTTAGAGTTCTTGAGAAGCTGCAC
	TCCTGTCAACAGTGCGGATGGCTCAGGCGGTGTGCGTTTGCTAGGAGCCACGGGGCCTGAGGAGCAAGCACAGATATTTA

Table S3 Summary of RNA-Seq analysis

Sample nane	Clean reads	Clean bases(G)	Q30(%)	GC content (%)
661w	42,663,288	5.37	94.60	53.22
Rp9-KI	38,561,492	4.85	93.81	52.25
Rp9-KO	38,834,744	4.88	94.13	52.93
